# Early Mother–Newborn Skin-to-Skin Contact at Term Birth and Early Neonatal Thermoregulation Under Routine Clinical Practice

**DOI:** 10.3390/medicina62010232

**Published:** 2026-01-22

**Authors:** Chia-Hui Liu, Sheng-You Su, Yuen-En Chang, Chia-Lung Shih

**Affiliations:** 1 Department of Nursing, Ditmanson Medical Foundation Chia-Yi Christian Hospital, Chiayi City 600, Taiwan; 02217@cych.org.tw (C.-H.L.); 06063@cych.org.tw (Y.-E.C.); 2 Clinical Research Center, Ditmanson Medical Foundation Chia-Yi Christian Hospital, Chiayi City 600, Taiwan; 15334@cych.org.tw

**Keywords:** skin-to-skin contact, neonatal thermoregulation, hypothermia, routine postbirth care, type of delivery

## Abstract

*Background and Objectives*: Early mother–newborn skin-to-skin contact (SSC) after birth is widely recommended to support neonatal physiological stabilization, including thermoregulation. Under routine clinical practice, however, SSC may be brief or interrupted, and its effectiveness in maintaining neonatal body temperature under such conditions is less well described. This study aimed to evaluate early neonatal temperature changes under routine post-birth care practices that included brief SSC followed by separation for incubation care. *Materials and Methods*: This retrospective cohort study included 620 term mother–infant dyads delivered at a single regional teaching hospital. Newborns were managed according to routine clinical practice and were allocated to either a brief early SSC group or a control group without SSC. SSC duration differed by mode of delivery (approximately 10 min after cesarean section and 20 min after vaginal birth). Infant body temperature was recorded at predefined time points from birth through early incubation care. Associations between temperature changes and clinical factors, including mode of delivery, gestational age, parity, and birth weight, were analyzed. *Results:* No significant difference was observed between the SSC and control groups in overall changes in infant body temperature from birth to the beginning of incubation care (*p* = 0.245). After one hour of incubation, mean body temperature was comparable between groups (*p* = 0.357). Within the SSC group, infant body temperature decreased significantly during the SSC period (change from birth: −0.68 °C ± 0.35 °C; *p* < 0.001). At the start of incubation care, a significantly lower proportion of infants in the SSC group (22%) had body temperatures below 36.5 °C compared to the control group (32%) (*p* = 0.018). Multivariable analysis identified mode of delivery, reflecting differences in post-birth care routines and SSC duration, as the only factor independently associated with temperature changes during SSC. *Conclusions:* Under routine clinical conditions, brief and interrupted SSC was associated with transient reductions in neonatal body temperature; however, brief SSC was associated with a lower proportion of hypothermia compared with immediate incubation care, suggesting that even short periods of SSC may support early neonatal thermoregulation.

## 1. Introduction

Skin-to-skin contact (SSC), defined as placing the naked newborn prone on the mother’s bare chest immediately after birth and covering the dyad with warm, dry textiles, is widely recommended as standard care for healthy newborns [1]. Extensive evidence has demonstrated that immediate and uninterrupted SSC supports neonatal physiological stabilization, including positively influence maternal–infant interaction 1 year later, thermoregulation, cardiorespiratory adaptation, breastfeeding initiation, and early mother–infant bonding [2,3,4]. Accordingly, the World Health Organization and multiple international guidelines recommend initiating SSC as soon as possible after birth and maintaining it continuously for at least 1 h in stable term and preterm newborns [5].

Recent obstetric literature has increasingly emphasized the concept of identifying and stratifying patients at higher risk in order to optimize perinatal care. In particular, the framework of the “ideal birth,” as described by Aquino et al., highlights how the recognition of clinical, intrapartum, and neonatal risk factors may allow clinicians to move beyond uniform care models and toward more individualized and preventive strategies [6]. Although this approach has been primarily applied to maternal outcomes, it may also be relevant to immediate postnatal care and early neonatal adaptation. Under routine clinical conditions, deviations from recommended standards—such as brief or interrupted SSC—may not affect all newborns equally. Evaluating early neonatal thermoregulation within a risk stratification perspective may therefore help to identify more vulnerable newborns and clarify how modifiable aspects of routine post-birth care influence early physiological stability.

However, recent evidence has highlighted important gaps between recommended SSC practices and routine clinical implementation. The updated Cochrane systematic review by Moore et al. emphasized substantial heterogeneity across SSC studies, largely attributable to variations in timing, duration, and continuity of SSC interventions [2]. Many included trials involved brief or interrupted SSC, particularly following cesarean delivery, and lacked detailed descriptions of routine post-birth care practices. As a result, the effects of SSC under real-world clinical conditions remain incompletely understood, despite strong evidence supporting its benefits under ideal circumstances.

Thermoregulation is a critical component of neonatal adaptation immediately after birth. Newborns are particularly vulnerable to heat loss during this period, and deviations from recommended thermal care practices may have important physiological implications. While uninterrupted SSC has been shown to support thermal stability [7], less is known about neonatal temperature changes when SSC is brief or interrupted and followed by early separation for incubation or observation, a pattern that remains common in routine clinical practice.

Despite strong evidence and clear recommendations, clinical practice in many birth settings does not fully adhere to uninterrupted SSC [7]. In routine care, SSC may be brief, delayed, or interrupted, particularly following cesarean delivery, and newborns may be separated early for observation or placed in incubators or warming areas. Such deviations from recommended practice may influence neonatal thermal stability; however, data describing neonatal temperature changes under these routine post-birth care conditions in term infants remain limited.

Therefore, the present study aimed to evaluate early neonatal body temperature changes under routine post-birth care conditions that included brief early SSC followed by early separation, compared with routine care without SSC. By focusing on real-world clinical practice rather than idealized SSC protocols, this study seeks to clarify how post-birth care routines influence early neonatal thermoregulation and to help explain heterogeneity observed in previous SSC studies.

## 2. Materials and Methods

### 2.1. Study Design and Setting

This retrospective cohort study was conducted at Ditmanson Medical Foundation Chia-Yi Christian Hospital, a regional teaching hospital in Taiwan. Medical records of term newborns delivered between January and December 2022 were reviewed. The study was approved by the institutional review board (IRB No. IRB2024054), and the requirement for informed consent was waived due to the retrospective nature of the study. Our hospital manages approximately 1000 deliveries per year. A retrospective study design was chosen to evaluate neonatal thermoregulation under routine clinical conditions, in which post-birth care practices—including the duration and continuity of SSC—were determined by institutional protocols and real-world clinical constraints rather than by a predefined research intervention. This design allowed inclusion of a large, unselected cohort of term newborns managed according to standard care, thereby enhancing the external validity of the findings and providing insight into neonatal temperature changes as they occur in everyday clinical practice.

### 2.2. Participants

Eligible participants were term mother–infant dyads admitted for childbirth during the study period. Newborns were excluded if their body temperature at the first recorded measurement after birth was <36.5 °C or >38.0 °C, or if birth weight was <2300 g, in accordance with local neonatal care protocols that prioritize intensive thermal management for very low birth-weight infants. The sample size was determined by the total number of eligible term mother–infant dyads delivered at the study center during the predefined study period. This approach was consistent with the retrospective nature of the study and allowed inclusion of all consecutive cases meeting the eligibility criteria, thereby minimizing selection bias and reflecting routine clinical practice at a regional teaching hospital.

### 2.3. Routine Post-Birth Care and Group Allocation

Post-birth care was determined by routine clinical practice and maternal preference when feasible. Newborns were allocated to either a brief early SSC group or a control group without SSC. In our institution, SSC during the early post-birth period is performed exclusively by the mother. Father SSC after cesarean section is not part of routine clinical practice and was not implemented during the study period. In this setting, SSC is implemented according to institutional protocols. As a result, the duration and continuity of SSC in this cohort were determined by routine clinical regulations, which led to brief and occasionally interrupted SSC rather than prolonged uninterrupted contact.

In the SSC group, newborns were placed in the prone position on the mother’s bare chest within 1 h after birth. The duration of SSC varied by mode of delivery, lasting approximately 20 min after vaginal delivery and 10 min after cesarean section, in accordance with institutional routines. Following completion of SSC, newborns were transferred to an incubation care area for further observation. For cesarean section deliveries, newborns were subsequently placed in a warming incubator and observed for one hour. In the control group, newborns were placed in a warming incubator immediately after birth and transferred directly to the incubation care area within 5 min, without SSC.

### 2.4. Thermal Care and Environmental Conditions

According to institutional routine practice, newborns were dried immediately after birth and covered with dry textiles during post-birth care. Specifically, amniotic fluid and blood were first wiped off using an initial layer of infant wrapping cloth, which was then removed and replaced with a dry wrap once wet. This was followed by umbilical cord clamping, measurement of infant body temperature and birth weight, and a routine physical examination to assess for any visible abnormalities. Delivery room and operating theater temperatures were maintained in accordance with hospital policy, typically ranging from 20 to 24 °C in the delivery room and from 23 to 25 °C in the SSC area. The incubation care area was equipped with warming devices in accordance with standard neonatal protocols, and ambient temperatures were maintained between 24 and 26 °C.

### 2.5. Temperature Measurement

Infant body temperature was measured using rectal temperature measurement with a standardized digital clinical thermometer (HCW, Foru International Co., Ltd., Taipei City, Taiwan) approximately 3–5 min after birth, in accordance with routine clinical practice. Temperature measurements were performed by trained nursing staff and documented in the medical record. In the SSC group, temperature was recorded at four predefined time points: (1) the first recorded measurement after birth, (2) immediately after completion of SSC, (3) at the beginning of incubation care, and (4) one hour after incubation care. In the control group, temperature was recorded at three time points: (1) the first recorded measurement after birth, (2) at the beginning of incubation care, and (3) one hour after incubation care.

### 2.6. Statistical Analysis

All statistical analyses were performed using SPSS (version 28.0; SPSS, Chicago, IL, USA). Continuous data are presented in terms of mean and SD values, whereas categorical data are presented in terms of frequency and percentage values. Post-SSC changes in infant body temperature were analyzed using a paired *t* test. The between-group difference in body temperature was analyzed using a two-sample t test. Between-group differences in categorical variables were analyzed using the chi-square test. Correlation analyses were performed through Pearson correlation analysis. Factors influencing infant body temperature were identified through multiple linear regression. A *p* value of <0.05 indicated significance.

## 3. Results

### 3.1. Study Population

**Figure 1:**
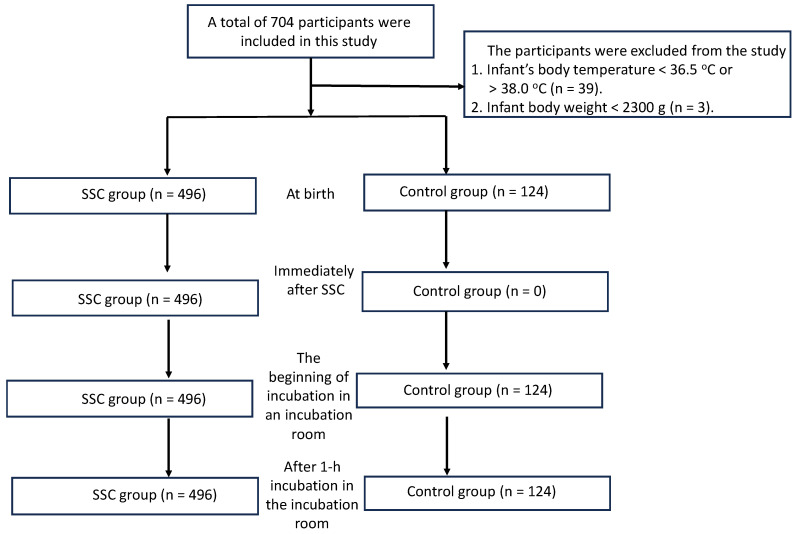
Flowchart depicting participant selection.

During the study period, a total of 704 mother–infant dyads delivered at term were initially identified from the hospital medical records. Newborns were subsequently excluded if the first recorded post-birth body temperature was <36.5 °C or >38.0 °C or if birth weight was <2300 g, in accordance with institutional neonatal care protocols that prioritize intensive thermal management for infants at risk of temperature instability. After application of these predefined exclusion criteria, 620 term newborns remained eligible and were included in the final analysis (Figure 1). Of the included newborns, 496 (80.0%) received brief early SSC following birth, whereas 124 (20.0%) were managed with routine post-birth care without SSC. Allocation to the SSC or control group was determined by routine clinical practice and maternal preference when feasible, rather than by study assignment. This cohort therefore represents an unselected population of term newborns managed under real-world post-birth care conditions.

### 3.2. Baseline Characteristics

Baseline maternal and neonatal characteristics are summarized in Table 1. Overall, the two groups were broadly comparable with respect to key demographic and neonatal variables. Parity and birth weight did not differ significantly between the SSC and control groups, indicating similar maternal obstetric profiles and neonatal size at birth. Gestational age at delivery was slightly but significantly higher in the SSC group compared with the control group (38.5 ± 1.02 vs. 38.0 ± 1.37 weeks; *p* < 0.001; Table 1). With regard to mode of delivery, vaginal birth occurred more frequently in the SSC group, whereas cesarean delivery was proportionally more common in the control group (74.2% vs. 60% vaginal delivery, respectively; *p* = 0.001; Table 1). At the first recorded post-birth measurement, mean neonatal body temperature was higher in the SSC group than in the control group (37.5 ± 0.33 °C vs. 37.3 ± 0.38 °C; *p* < 0.001; Table 1). However, following one hour of incubation care, no significant difference in mean body temperature was observed between the two groups (37.0 ± 0.32 °C in the SSC group vs. 37.0 ± 0.32 °C in the control group; *p* = 0.357), suggesting convergence of thermal status after standardized incubation.

### 3.3. Changes in Infant Body Temperature over Time

**Figure 2:**
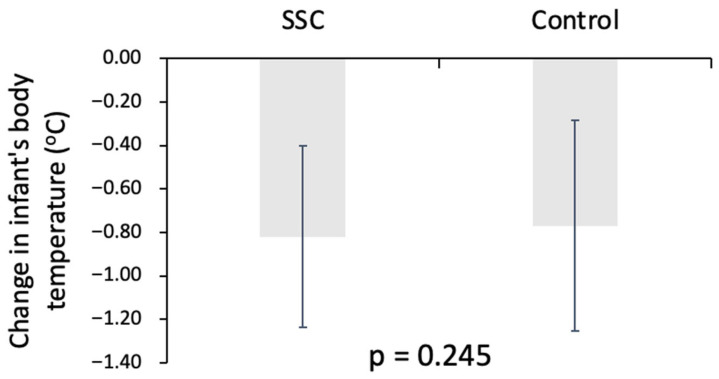
Changes in infant body temperature from birth to beginning of incubation in SSC and control groups.

**Figure 3:**
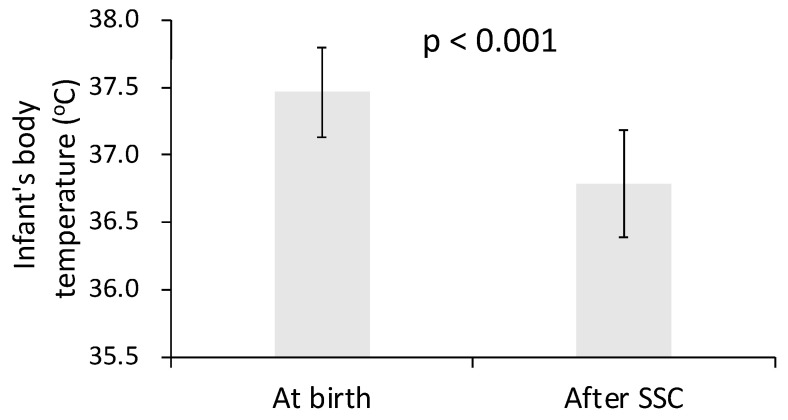
Infant body temperature at birth and immediately after SSC.

Changes in neonatal body temperature over time are illustrated in Figure 2 and Figure 3. When comparing the overall change in body temperature from the first recorded post-birth measurement to the beginning of incubation care, no significant difference was observed between the SSC and control groups (*p* = 0.245), indicating similar net temperature changes across this early postnatal interval regardless of exposure to SSC (Figure 2). Following one hour of incubation care, mean neonatal body temperature did not differ significantly between the two groups (*p* > 0.05), suggesting convergence of thermal status after standardized incubation. In contrast, within-group analysis of the SSC cohort demonstrated a significant decrease in body temperature during the SSC period itself. Specifically, infant body temperature declined from the first recorded post-birth measurement to the completion of SSC, with a mean change of −0.68 ± 0.35 °C (*p* < 0.001) (Figure 3). This finding indicates that, under routine clinical conditions with brief and interrupted SSC, a measurable transient reduction in neonatal body temperature occurred during the SSC interval.

### 3.4. Incidence of Hypothermia

Hypothermia was defined a priori as a rectal body temperature < 36.5 °C. In the SSC group, 61 of 496 infants (15.3%) met the criterion for hypothermia at the end of the SSC period, indicating that a subset of newborns experienced temperatures below the threshold during this brief immediate post-birth contact. At the beginning of incubation care (i.e., immediately after transfer to the incubation care area), hypothermia was observed in 22% of infants in the SSC group compared with 32% in the control group, demonstrating a significantly lower proportion of hypothermia among infants who received brief SSC than among those who were separated immediately and placed in a warming incubator after birth (*p* = 0.018). These findings indicate that although hypothermia occurred during the early postnatal period in both groups, brief SSC was associated with a lower rate of hypothermia at the transition into incubation care.

### 3.5. Factors Associated with Changes in Body Temperature

Correlation analyses showed no significant associations between temperature change during SSC and parity or birth weight (Table 2). Gestational age was weakly correlated with temperature change (Table 2). We found no significant difference in temperature change during SSC between low-birth-weight and normal-birth-weight newborns (Table 3). However, vaginal delivery was associated with a significantly greater decrease in temperature during SSC compared with cesarean section (Table 3). In multivariable linear regression analysis adjusting for gestational age, parity, and birth weight, mode of delivery was the only variable independently associated with temperature change during the SSC period (Table 4).

## 4. Discussion

In this retrospective cohort study conducted under routine clinical practice, we examined early neonatal body temperature trajectories in term infants exposed to brief post-birth SSC followed by early separation for incubation care. Our results showed that neonatal body temperature declined significantly during the SSC period, indicating a transient thermal decrease associated with brief and interrupted SSC under routine care conditions. Despite this initial decline, infants who received brief SSC did not exhibit a higher incidence of hypothermia compared with those managed with immediate incubation care. Notably, at the initiation of incubation care, the proportion of infants with body temperatures below 36.5 °C was significantly lower in the SSC group than in the control group, and mean body temperature did not differ between groups after one hour of incubation. These findings suggest that, even when SSC is brief and interrupted, it may confer partial thermal protection during the early postnatal period compared with immediate separation alone.

Early and uninterrupted SSC has been consistently shown to support neonatal thermoregulation in both term and preterm infants when appropriate thermal protection measures—such as immediate drying, adequate covering, and maintenance of a warm environment—are applied [2,8]. Under these optimal conditions, SSC facilitates heat transfer from the mother to the newborn, reduces heat loss through evaporation and convection, and promotes physiological stability during the critical transition from intrauterine to extrauterine life. Accordingly, international guidelines, including those from the World Health Organization, recommend the initiation of SSC as soon as possible after birth and its continuous maintenance for at least one hour as a cornerstone of high-quality neonatal care [1,5].

The present study does not challenge this well-established evidence base. Rather, it addresses a complementary and clinically relevant question by examining neonatal temperature changes under routine clinical conditions in which SSC is brief and interrupted. Such care patterns, which often result from institutional protocols, environmental constraints, and competing clinical priorities, remain common in many birth settings. By focusing on these real-world practices, our findings provide additional context for interpreting the variability observed across SSC studies [2] and help to bridge the gap between guideline recommendations and everyday clinical implementation.

Evidence from trials with longer and more continuous SSC generally shows improved thermal outcomes and lower hypothermia risk. For example, Nimbalkar et al. found that providing early SSC for prolonged periods (with minimal interruption) markedly reduced hypothermia incidence compared with conventional care [8]. In contrast, our cohort reflects a real-world model of brief and interrupted SSC followed by early separation, in which a transient decline in temperature occurred during SSC, yet the proportion of hypothermia at the initiation of incubation care was lower than with immediate incubator care. Although brief SSC may provide thermal benefits, it remains unclear whether its effects are comparable to those achieved with continuous and uninterrupted SSC lasting more than one hour.

The observed reduction in body temperature during the SSC period in our cohort is likely attributable to a combination of neonatal physiological vulnerability and post-birth handling routines. Newborns are particularly susceptible to heat loss through evaporation, convection, conduction, and radiation, especially during the immediate postnatal period [9]. Early separation, transfer between care environments, and variations in drying, covering, and environmental temperature may all contribute to transient decreases in body temperature. These factors are related to clinical practice rather than the SSC intervention itself. Our results showed that infants delivered vaginally experienced significantly greater temperature loss than those delivered by cesarean section. This difference may be attributable to variations in perinatal care processes between delivery modes. Vaginal delivery is often associated with longer exposure to the ambient delivery room environment, increased evaporative heat loss due to amniotic fluid, and delays in initiating effective thermal protection measures. In contrast, cesarean delivery typically occurs in a more controlled thermal environment, with earlier drying, wrapping, and application of standardized warming interventions, which may collectively reduce neonatal heat loss during the immediate postnatal period.

Although a proportion of infants exhibited body temperatures below 36.5 °C during the early postnatal period, the incidence of hypothermia at the start of incubation care was lower in the brief SSC group than in the control group. This finding suggests that even short periods of SSC, when combined with routine thermal care, may contribute to early thermal stabilization compared with immediate separation alone. The Cochrane review on early SSC concluded that SSC probably helps newborns maintain stable body temperature and supports physiologic transition after birth [2]. In addition, studies comparing different ward routines have shown that post-birth care pathways and handling practices materially shape neonatal temperature trajectories, and SSC may reduce temperature-related stress when integrated into care routines [10]. Together, these findings provide a plausible explanation for our observation that even a short exposure to SSC—despite being interrupted—may confer partial protection against hypothermia compared with immediate separation alone. However, the relatively high overall incidence of hypothermia observed in both groups likely reflects early interruption of SSC and the limitations of routine thermal protection measures rather than inherent risks associated with SSC.

Mode of delivery emerged as the only factor independently associated with temperature changes during the SSC period. This association should not be interpreted as a physiological effect of vaginal or cesarean delivery per se. Instead, mode of delivery likely served as a proxy for differences in post-birth care routines, including SSC duration, environmental conditions, and handling practices. In our institution, SSC duration was shorter following cesarean section than vaginal delivery, which may have influenced observed temperature changes.

The findings of this study underscore the importance of adherence to recommended practices for uninterrupted SSC and comprehensive thermal protection during the immediate postnatal period. From a clinical perspective, optimizing environmental temperature, minimizing unnecessary separation, and ensuring adequate drying and covering are essential to support neonatal thermoregulation, particularly when SSC is initiated.

Several limitations should be acknowledged. First, the retrospective design restricted the availability and granularity of certain clinical variables, including the exact timing of temperature measurements, the duration of individual handling steps, and detailed documentation of thermal protection measures such as drying techniques, type and timing of covering, and use of supplemental warming devices. Variability in environmental conditions across delivery rooms, operating theaters, and incubation areas could not be fully captured, which may have contributed to unmeasured confounding in observed temperature changes. Second, this study was conducted at a single regional teaching hospital, and post-birth care practices—including SSC duration, workflow, and environmental management—are influenced by institution-specific protocols. Therefore, the findings may not be directly generalizable to centers with different staffing models, facility layouts, or adherence to uninterrupted SSC recommendations. Third, the SSC implemented in this cohort was brief and interrupted by early separation for routine care, which differs substantially from international guidelines advocating early and continuous SSC for at least one hour. As a result, the observed temperature changes reflect the effects of brief, real-world SSC rather than the physiological impact of uninterrupted SSC. Caution is therefore warranted when extrapolating these findings to settings where continuous maternal SSC is consistently achieved. Finally, the absence of certain potentially relevant variables—such as labor stimulation or induction, neonatal acid–base status, and alternative SSC strategies (e.g., paternal SSC)—limited the ability to explore additional mechanisms influencing neonatal thermoregulation. These factors should be considered in future prospective studies designed to more comprehensively evaluate thermal adaptation during the immediate postnatal period.

Future research should build on these findings by prospectively evaluating neonatal thermoregulation under standardized yet pragmatic post-birth care protocols. In particular, studies designed to compare uninterrupted versus brief or interrupted SSC, while carefully documenting timing, duration, and continuity of SSC, would help to clarify dose–response relationships between SSC exposure and thermal stability. Detailed recording of thermal protection measures (e.g., drying practices, covering, environmental temperature, and timing of transfers) would further improve understanding of modifiable contributors to early heat loss. Given the observed association between mode of delivery and temperature changes, future studies should also explore delivery-specific post-birth care pathways, with attention to how workflow, environment, and handling differ between vaginal and cesarean births. When maternal SSC is not feasible, alternative strategies—such as paternal SSC or enhanced thermal protection during early separation—warrant systematic evaluation. In addition, incorporation of physiological indicators beyond body temperature, including cardiorespiratory parameters and metabolic markers, may provide a more comprehensive assessment of neonatal adaptation. Finally, multicenter studies across diverse clinical settings would enhance generalizability and help identify institution-level practices that best support early neonatal thermoregulation under real-world conditions.

## 5. Conclusions

In this retrospective cohort study conducted under routine clinical practice, we examined early neonatal body temperature changes associated with brief, early post-birth SSC followed by early separation for incubation care. Our findings demonstrated that a transient reduction in neonatal body temperature occurred during the SSC period; however, brief SSC was not associated with a higher risk of hypothermia compared with routine care without SSC. Importantly, a lower proportion of infants in the SSC group had body temperatures below 36.5 °C at the initiation of incubation care. These results do not contradict the well-established benefits of early and uninterrupted SSC for neonatal thermoregulation. Rather, they highlight how deviations from recommended SSC practices—specifically brief duration and early interruption under routine clinical conditions—may influence early thermal stability. Our findings suggest that observed temperature changes are more strongly related to post-birth care routines and handling practices than to the SSC intervention itself. Differences in temperature changes were primarily associated with mode of delivery, which reflected variations in post-birth care routines and SSC duration rather than physiological effects of the delivery mode itself. These findings underscore that neonatal thermoregulation during the early postnatal period is strongly influenced by clinical handling, environmental conditions, and continuity of care.

## Figures and Tables

**Table 1:** Major characteristics of study cohort.

	SSC	Control Group	*p* Value
	*n* = 496	*n* = 124	
Parity	1.78 ± 0.89	1.87 ± 0.99	0.299
Gestational age (weeks)	38.5 ± 1.02	38.0 ± 1.37	<0.001
Type of delivery			
Cesarean section	128 (25.8%)	50 (40%)	0.001
Vaginal birth	368 (74.2%)	74 (60%)	
Birth weight (g)	3068 ± 332	3185 ± 2143	0.543
Infant body temperature at birth (°C)	37.5 ± 0.33	37.3 ± 0.38	<0.001

SSC, skin-to-skin contact.

**Table 2:** Correlations between infant body temperature changes during SSC and various factors.

		Parity	Gestational Age	Birth Weight
Changes in body temperature during SSC	r	0.038	−0.147	0.080
	*p* value	0.404	0.001	0.076
	*n*	496	496	496

SSC, skin-to-skin contact.

**Table 3:** Factors influencing infant body temperature changes during SSC.

	*n*	Mean	SD	*p* Value
Infant’s body weight				
Low birth weight (<2500 g)	23	−0.79	0.38	0.132
Normal weight	473	−0.68	0.35	
Type of delivery				
Cesarean section	128	−0.49	0.27	<0.001
Vaginal birth	368	−0.75	0.36	

**Table 4:** Results of multiple regression performed to identify factors significantly influencing changes in infant body temperature during SSC.

	β	95% CI of β	*p* Value	VIF
Gestational age	−0.021	−0.051 to 0.009	0.176	1.088
Type of delivery (reference: vaginal birth)	0.248	0.177 to 0.318	<0.001	1.088

VIF, variance inflation factor.

## Data Availability

The datasets generated and/or analyzed during the current study are not publicly available due to ethical restrictions involving human participant privacy. The data supporting the findings of this study are available from the corresponding author upon reasonable request.

## Abbreviations

SSC, skin-to-skin contact; WHO, World Health Organization; IRB, Institutional Review Board; SD, standard deviation.
